# A Fiber-Coupled Scanning Magnetometer with Nitrogen-Vacancy Spins in a Diamond Nanobeam

**DOI:** 10.1021/acsphotonics.3c00259

**Published:** 2023-05-25

**Authors:** Yufan Li, Fabian A. Gerritsma, Samer Kurdi, Nina Codreanu, Simon Gröblacher, Ronald Hanson, Richard Norte, Toeno van der Sar

**Affiliations:** † Department of Quantum Nanoscience, Kavli Institute of Nanoscience, Delft University of Technology, Delft 2628CJ, The Netherlands; ‡ Department of Precision and Microsystems Engineering, Faculty of Mechanical, Maritime and Materials Engineering, Delft University of Technology, Delft 2628CD, The Netherlands; § QuTech and Kavli Institute of Nanoscience, Delft University of Technology, Delft 2628CJ, The Netherlands

**Keywords:** nitrogen-vacancy magnetometry, quantum sensing, diamond nanophotonics, diamond nanobeam, fiber-coupled sensor

## Abstract

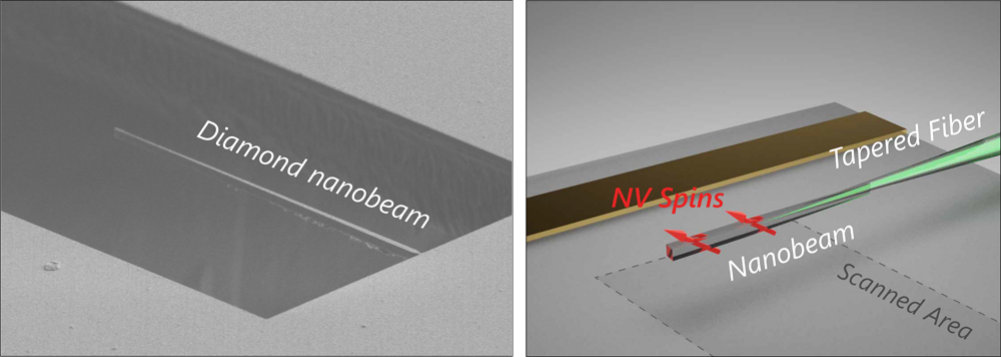

Magnetic imaging with nitrogen-vacancy (NV) spins in diamond is becoming an established tool for studying nanoscale physics in condensed matter systems. However, the optical access required for NV spin readout remains an important hurdle for operation in challenging environments such as millikelvin cryostats or biological systems. Here, we demonstrate a scanning-NV sensor consisting of a diamond nanobeam that is optically coupled to a tapered optical fiber. This nanobeam sensor combines a natural scanning-probe geometry with high-efficiency through-fiber optical excitation and readout of the NV spins. We demonstrate through-fiber optically interrogated electron spin resonance and proof-of-principle magnetometry operation by imaging spin waves in an yttrium-iron-garnet thin film. Our scanning-nanobeam sensor can be combined with nanophotonic structuring to control the light–matter interaction strength and has potential for applications that benefit from all-fiber sensor access, such as millikelvin systems.

## Introduction

The nitrogen-vacancy (NV) lattice defect in diamond has emerged as a powerful magnetic-field sensor. High-fidelity microwave control and optical readout of the NV spin^1−3^ over a wide range of conditions has enabled applications in condensed matter physics,^4^ chemistry,^5^ biology,^6,7^ and geoscience.^8^ In particular, scanning-probe magnetometry based on individual NV spins in diamond nanotips has provided imaging of spins and currents in materials with spatial resolution down to ∼50 nm.^9−11^

An important challenge for the application of scanning-probe NV magnetometry in advanced environments such as millikelvin cryostats is the required optical access to the NV spins. Free-space optical access leads to additional heat load and increased complexity of cryostat design. A potential way to preclude the need for free-space optical access is to realize fiber-based scanning-NV sensors.^12,13^ Here, we demonstrate a new scanning-NV sensor based on a tapered diamond nanobeam that is optically coupled to, and manipulated with, a tapered optical fiber. Such fiber-based NV nanobeam sensors could facilitate implementation in low-temperature setups, while benefiting from the potentially near-perfect optical coupling efficiency between fiber and nanobeam.^14−16^ Moreover, nanobeams are excellently suited for nanophotonic structuring,^17,18^ which could enable high-efficiency, resonant optical addressing of embedded NV centers or other group-IV color centers^19^ by incorporating photonic crystals.

**Figure 1:**
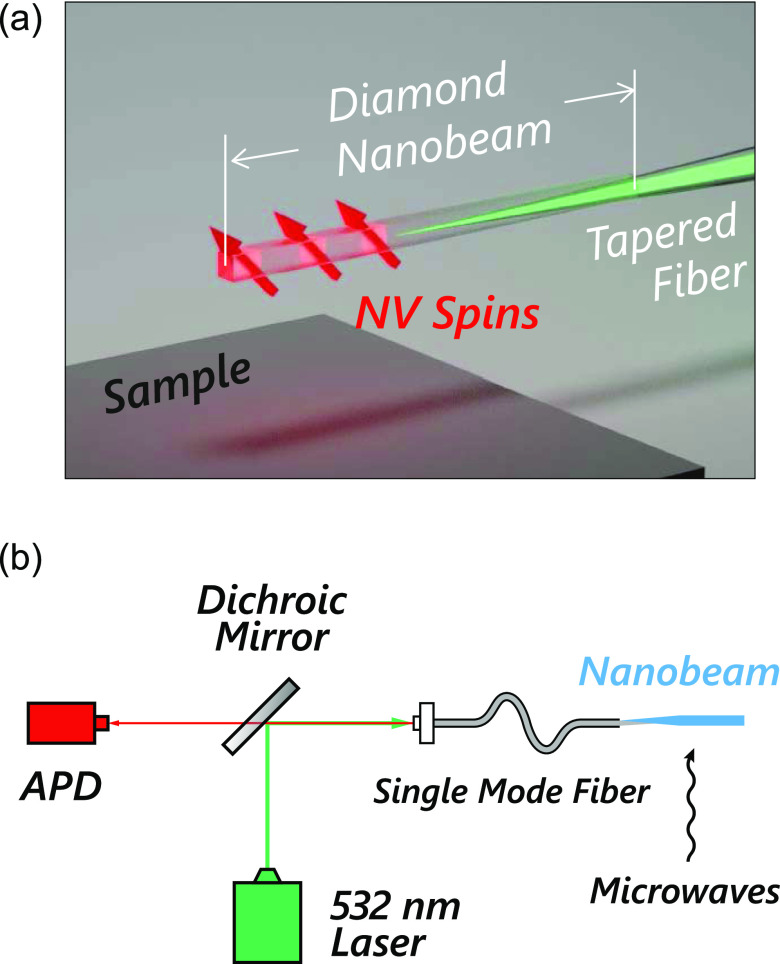
Magnetometry based on nitrogen-vacancy spins in fiber-coupled diamond nanobeams. (a) Schematic illustration of the technique: A diamond nanobeam with an ensemble of NV spins is optically coupled to a tapered fiber. The fiber both guides the excitation laser to the NVs and collects the NV photoluminescence, resulting in a scanning-probe magnetometer that does not require free-space optical access. Scanning the nanobeam parallel to the sample surface enables magnetic imaging with high resolution in the direction perpendicular to the beam axis. (b) Simplified scheme of the optics used to excite and read out the NV photoluminescence. A 532 nm laser excitation is coupled into the fiber that delivers the light to the NV centers in the diamond nanobeam. The resulting NV photoluminescence is collected through the same fiber, separated from the excitation light by a dichroic mirror, and detected by an avalanche photodiode (APD).

We fabricate the diamond nanobeams using nanofabrication recipes developed in refs (18 and 20−22). The key advance we present here is the ability to break off and attach individual tapered diamond nanobeams to nanoscale-tapered optical fibers and use these nanobeam sensors for scanning NV magnetometry (Figure 1a). We break a beam off the bulk diamond by pushing on it with the fiber, after which the beam and the fiber remain attached, presumably by van der Waals forces.^23,24^ By designing nanobeams with large aspect ratios and nanometer-scale holding bars, we overcome the large yield strength and the strong elastic deformation of diamond nanostructures caused by the applied force, which enables us to break off the beam while simultaneously attaching it to the fiber. As a proof of principle, we demonstrate through-fiber optical interrogation of an NV-nanobeam sensor, characterize its photon collection efficiency, and demonstrate its imaging capability by visualizing spin waves in a thin film of yttrium-iron garnet (YIG).^11,25^

## Results and Discussion

**Figure 2:**
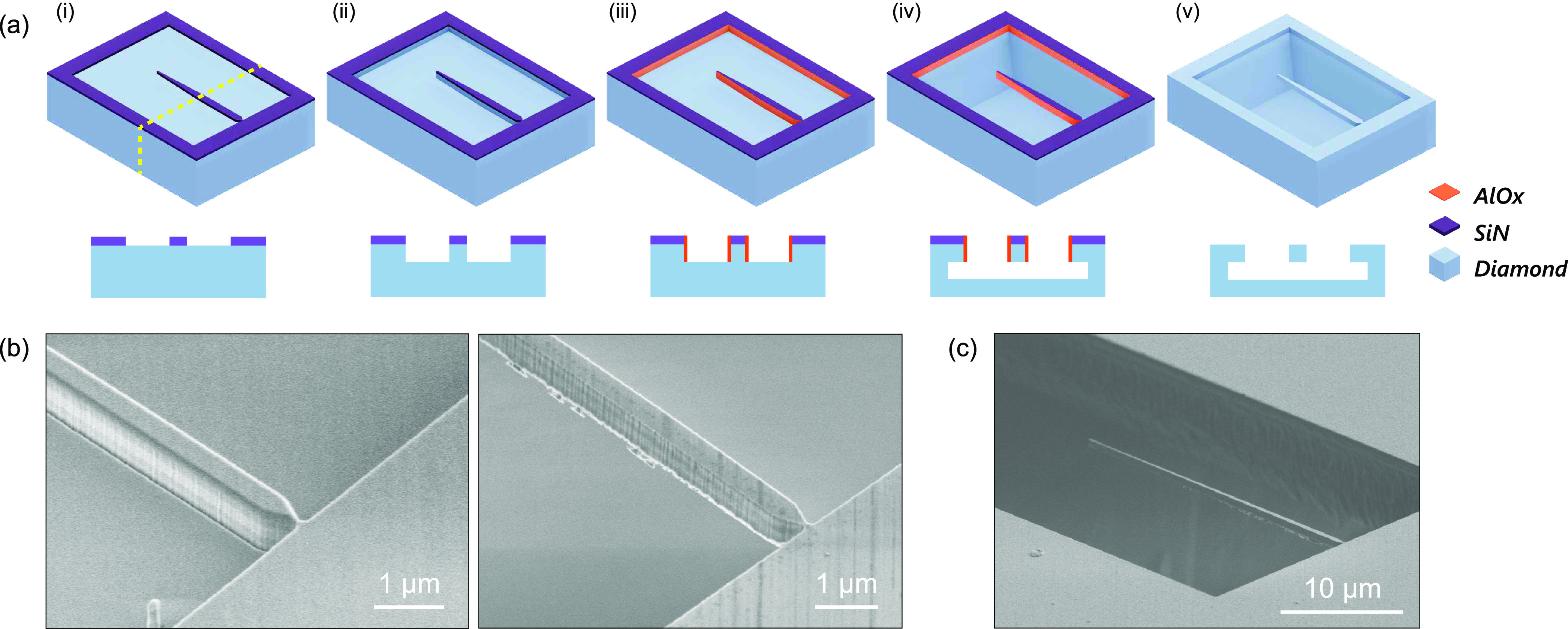
Fabrication of large-aspect-ratio diamond nanobeams that enable breaking-off and fiber coupling. (a) Fabrication flow.^20^ (i) A Si_3_N_4_ hard mask (∼200 nm thickness, purple) is fabricated on a diamond substrate (blue) using electron-beam lithography and anisotropic CHF_3_/O_2_ reactive ion etching (RIE). (ii) An anisotropic O_2_ RIE process defines the nanobeam sidewalls in the diamond. (iii) A ∼20 nm layer of Al_2_O_3_ (orange) is grown by atomic layer deposition (ALD) to protect the nanobeam sidewalls during the subsequent undercut step. An anisotropic BCl_3_/Cl_2_ RIE step removes the Al_2_O_3_ on the horizontal surfaces. (iv) An isotropic O_2_ RIE process undercuts the diamond nanobeam. (v) Removal of all masks with hydrofluoric (HF) acid. The schematics beneath each panel show corresponding cross-sectional views, marked with the yellow dashed line in panel (i). (b) Scanning electron microscope (SEM) images of representative nanobeams during fabrication (zoomed in to show the connection point), taken at stages illustrated in panels (ii) (left) and (v) (right) in (a). (c) SEM image of a 40 μm long diamond nanobeam after the fabrication.

Efficient optical coupling to the fiber requires tapered diamond nanobeams with nanoscale widths.^15^ We fabricate these beams out of a single-crystal diamond chip using the procedure demonstrated in ref (22) (Figure 2). We first deposit a 200 nm thick Si_3_N_4_ mask onto the diamond using plasma-enhanced chemical vapor deposition (PECVD). We then use e-beam lithography and reactive ion etching (RIE) with a CHF_3_/O_2_ plasma to pattern the beams and their holding bars (“tethers”) on the Si_3_N_4_ hard mask. An anisotropic, inductively coupled plasma (ICP) RIE with O_2_ transfers the patterns from the hard mask to the diamond substrate (Figure 2b). Using atomic layer deposition (ALD) to deposit 20 nm of Al_2_O_3_,^18^ we create a conformal layer that protects the vertical sidewalls during the subsequent undercut. An anisotropic ICP-RIE with BCl_3_/Cl_2_ removes the Al_2_O_3_ from the horizontal diamond surfaces while leaving the vertical beam sidewalls protected. Finally, we undercut the nanobeams with a quasi-isotropic O_2_ ICP-RIE and remove the masks with hydrofluoric acid (HF), leaving free-hanging diamond nanobeams (Figure 2c).

**Figure 3:**
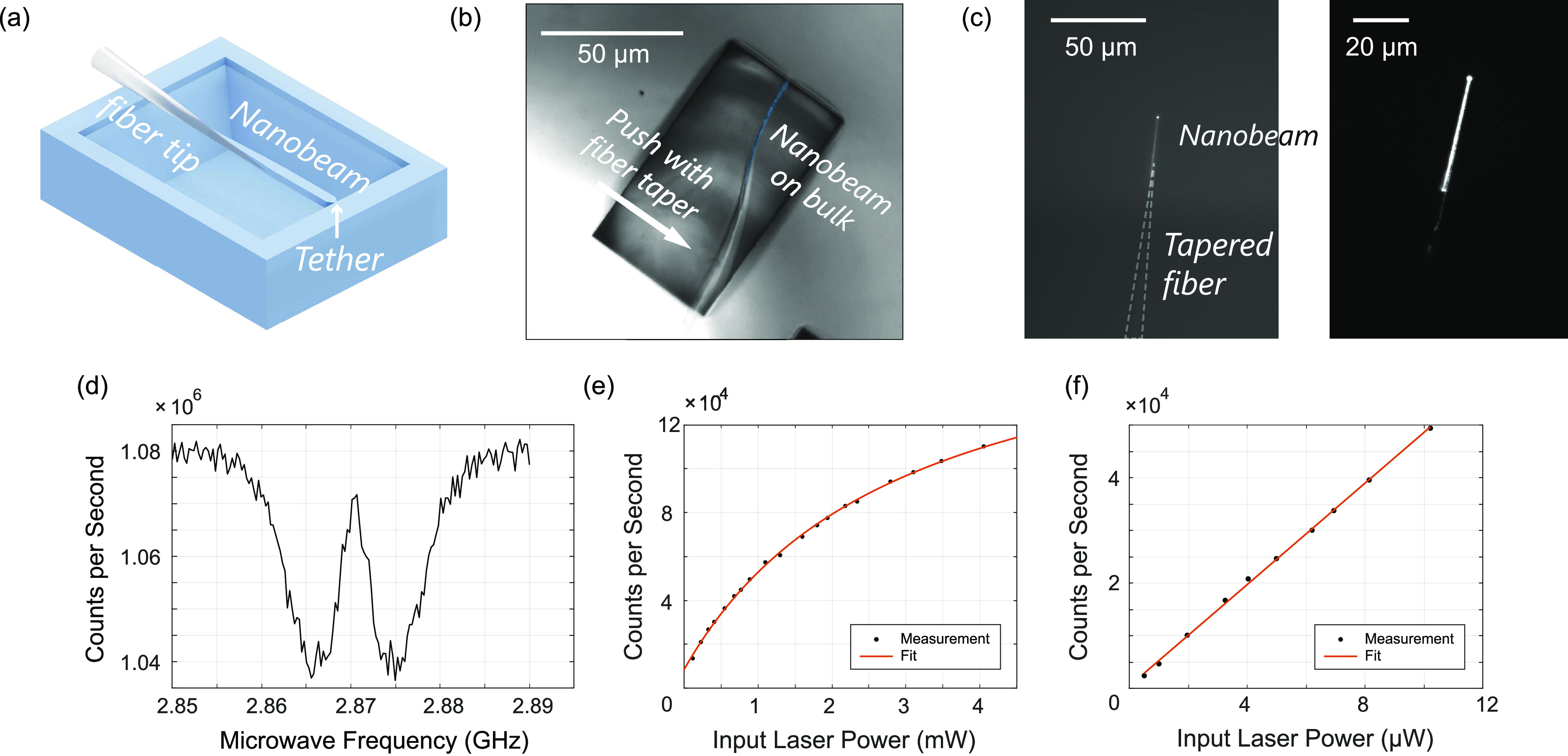
Assembly and characterization of a fiber-coupled diamond nanobeam sensor. (a) Schematic illustration of a tapered fiber brought into contact with a nanobeam. (b) Microscope image of the fiber pushing sideways on the nanobeam (artificially colored in blue) to break the ∼70 nm-wide connection to the diamond chip. Both beam and fiber bend strongly before breaking. (c) After breaking off, the beam sticks to the fiber (outlined with the dashed line). Through-fiber green-laser excitation causes the bright NV photoluminescence visible in this camera image. The image is taken with a 650 nm long-pass filter to block the excitation light. Right panel is a zoom-in view and with higher excitation power, highlighting the NV photoluminescence in the beam. (d) NV electron spin resonance (ESR) measurement at zero magnetic field, measured using the setup depicted in Figure 1b. Optical excitation power: 30 nW. (e) NV photoluminescence vs optical excitation power, fitted with eq 1 (orange curve). The count rate on the *y*-axis is after neutral-density (ND) filtering by a factor of 6 × 10^6^ (so that the measured photoluminescence rate stays within the measurement range of the APD). (f) Characterization of fiber autoluminescence in our experiment. Linear fit gives an autoluminescence rate of Γ_fiber_ = 4.8 × 10^3^ s^–1^ μW^–1^.

To couple the nanobeams to a tapered optical fiber (S630-HP, tapered by HF pulling, see Methods), we mount the nanobeam chip on a 3-axis slip-stick positioner (Mechonics MX-35). Monitoring through a microscope objective (Mitutoyo M Plan Apo HR 50×), we push the fiber against the nanobeam by moving the stage perpendicularly to the beam until the connection point breaks and the nanobeam sticks to the fiber. The sticking is presumably due to van der Waals force. The process and end result are illustrated in Figure 3.

Compared to similar strategies of picking up nanophotonic structures made from other materials, such as Si or SiN,^23,24,26^ the main challenge lies in the significantly larger yield strength of diamond compared to glass.^27^ Also, single crystal diamond on the nanoscale is known to exhibit large elastic deformation before fracturing when pressure is applied,^28^ as we also observe while pushing on the beam with the fiber in Figure 3b. The resulting abrupt motion when the beam breaks makes it challenging to stick the beam to the optical fiber.

To overcome this challenge, we found it crucial to design beams that are at least 30 μm in length, which also ensures the adiabatic change of the effective refraction index according to ref (15). We also minimize the width of the tether by fabricating an array of devices with varying tether widths, and use the beams with the thinnest tethers that survived the fabrication process. Furthermore, the area of the open region around the beam should be large enough (in our design 70 μm × 40 μm) to allow for the beam displacement during the breaking process. With these design implementations, we are able to apply a large enough torque on the tether to break the nanobeam off the bulk with a tapered optical fiber, and couple the beam to the fiber in the same process. We find tether widths of 60–80 nm to be optimal, where around 60% of the beams remain attached to the bulk after the undercut and subsequent acid cleaning, and can be picked up by the tapered fiber with a success rate of 50% (five out of ten beams).

We demonstrate through-fiber optical excitation and readout of an ensemble of NV centers in a diamond nanobeam using the setup depicted in Figure 1b. Our nanobeams (fabricated using element-six DNV-B14 diamond) have an estimated NV concentration of 4.5 ppm,^29^ corresponding to *N* ≈ 4.5 × 10^6^ NVs per nanobeam (40 μm long, maximum cross section 0.5 × 0.5 μm^2^ and tapered down to ∼0.1 × 0.5 μm^2^ over 37 μm length). We apply microwaves (Windfreak SynthHD) through a coplanar waveguide to drive the electron-spin resonance (ESR) of the NV centers. Figure 3d shows a characteristic ESR spectrum measured through-fiber from our device, where the dips result from the microwave-driven transition between NV spin states |*m*_*S*_ = 0⟩ → |*m*_*S*_ = ±1⟩. Due to the high NV density, we record the ESR signal with only 30 nW of excitation power. Following the methodology in ref (30), we estimate from the ESR spectrum the shot-noise limited magnetic field sensitivity to be 4.2(3) μT/Hz^1/2^. This value is also limited by the collection efficiency of our optical setup, illustrated in the Supporting Information.

Comparing the low-power NV photoluminescence rate of 3.7 × 10^7^ s^–1^ μW^–1^ (Figure 3d) to the independently measured fiber autolunimescence rate of 4.8 × 10^3^ s^–1^ μW^–1^ (Figure 3f) shows that our signal is dominated by the NV photoluminescence, with a signal-to-background ratio of 8 × 10^3^. By normalizing the photon count in Figure 3d to the total number of NVs *N*, we estimate the collected photoluminescence rate of a single NV center to be Γ_NV_/*N* = 8.1 s^–1^ μW^–1^ ≪ Γ_fiber_. Therefore, in order to achieve efficient single NV readout where Γ_NV_/*N* ∼ Γ_fiber_, further effort is needed on both reducing the fiber autoluminescence and increasing the NV photon collection efficiency.

To estimate the photon coupling efficiency of the nanobeam–fiber interface η_nf_, we use two approaches. In the first, we characterize the saturation of the NV photoluminescence as a function of the optical excitation power *P*. Assuming a simple two-level model for the NV photodynamics, the NV photoluminescence is limited by the NV’s spontaneous emission rate γ = 1/(13 ns).^31^ As such, the photon count rate Γ detected by our avalanche photodiode (APD, Laser Component COUNT-500N-FC) can be described by
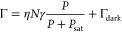
1Here, η is the fraction of total number of photons emitted by the NVs that is detected by our APD, *P*_sat_ is the optical saturation power,^30^ and Γ_dark_ is a power-independent background rate (including e.g. APD dark counts). Because of the strong NV luminescence (Figure 3e,f), we can omit the contribution of fiber autoluminescence and fit eq 1 to the data in Figure 3e. We extract η = 5.0(2) × 10^–10^. Writing η = η_ND_η_D_η_nf_, where η_ND_ = 1.6 × 10^–7^ is the neutral-density (ND) filtering factor and η_D_ = 3.5 × 10^–2^ is the optical efficiency of the other parts of our setup (characterized separately, see SI), we extract the photon coupling efficiency at the fiber–nanobeam interface η_nf_ = 8.6(4)%. We note that the relatively small error here derives from the fit uncertainty of η. However, other systematic uncertainties are likely to play a more important role. For example, the two-photon-induced ionization of the NV centers to the neutral charge state could affect the detected photon rate due to the different spectrum of NV^0^ centers.^32,33^ We observe an increased contribution of NV^0^ with increasing excitation power in the photoluminescence spectra of our device (Figure S3 of the SI). We therefore use a second approach to estimate η_nf_.

In the second approach, we estimate η_nf_ from the detected NV photoluminescence using a literature value for the NV’s absorption cross section σ_NV_ = 3.1(8) × 10^–21^ m^2^ for 532 nm laser excitation.^34^ Far below optical saturation, the detected NV photoluminescence is given by (see SI for detailed explanation)
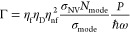
2where σ_mode_ is the cross-sectional area of the optical mode in the nanobeam, *N*_mode_ is the number of NV centers within the optical mode volume, η_f_ is the coupling efficiency of the excitation laser into the fiber, ω is the frequency of our 532 nm laser and *ℏ* is the Planck’s constant. Furthermore, we assumed that the coupling of the green laser from the fiber into the nanobeam is also given by η_nf_. In contrast with the first approach, this approach does not require saturating the NV photoluminescence response and can thus be conducted at very low (nW) laser power. This reduces the potential influence of two-photon-induced ionization of the NV centers to the neutral charge state.^32,33^ Assuming the mode is perfectly confined within the nanobeam, we take *N*_mode_/σ_mode_ ≈ *N*/σ_beam_, where σ_beam_ = 0.25 μm^2^ is the cross-sectional area of the nanobeam. From the measured Γ = 1.1 × 10^6^ s^–1^ at *P* = 30 nW, we can extract η_nf_ = 14(2)%, similar to the value found using the first approach. Combined with the estimated coupling efficiencies of 4 other nanobeam-fiber sensors (data presented in the SI), we obtain an average collection efficiency of 14% and a statistically estimated standard deviation of 4%.

We expect the found values for η_nf_ to be conservative estimates of the nanobeam–fiber coupling efficiencies due to the assumptions that all NV-emitted photons are radiated into the beam and toward the nanobeam-fiber interface, and because our two-level model neglects the nonradiative decay path via the singlet state that reduces the total photon emission rate.^35^ Also, since the optical lifetime of NV centers depends on the electromagnetic environment, the lifetime of NV centers in our nanobeams could be longer than that of NV centers in bulk diamond due to the lower refractive index of air.^36^ By using the bulk lifetime we obtain an estimation of the collection efficiency on the conservative side.

Compared to the state-of-the-art η_nf_ > 90% reported in refs (15 and 16) for single-wavelength (sub-nm spectral width) readout, an important difference in our device is the wide-band spectrum (bandwidth ∼ 200 nm) of the collected NV photoluminescence. Also, the precise alignment of the fiber tip required to optimize the coupling efficiency is affected by the abrupt motion of the nanobeam when the tether breaks: From a through-fiber measurement of the NV photoluminescence of a beam that is still attached to the bulk diamond (SI), we estimate using the absorption cross section method that η_nf_ = 19(3)%. Other factors that reduce the efficiency include the roughness on the sidewalls and bottom side of the nanobeams, which can be improved by optimizing the fabrication process, for instance by ion-based polishing of the nanobeam sidewall^37^ or improved diamond etching.

Considering the above, our estimated nanobeam–fiber coupling efficiency is remarkably high, paving the way for high-efficiency, ensemble-based NV sensing. However, the fiber autoluminescence would still exceed the single-NV photoluminescence by about an order of magnitude even in the limit η_nf_ → 1 (according to eq 2, Γ_NV,max_/*N* = 4.1 × 10^2^ s^–1^ μW^–1^). This indicates that single-NV sensing will only be possible if the fiber autoluminescence can be reduced, for example by incorporating hollow-core photonic crystal fibers that produce less fluorescence, following the method reported in ref (38), where the hollow-core fiber is spliced onto a short section of normal fiber that can be tapered and coupled to the nanobeam.

**Figure 4:**
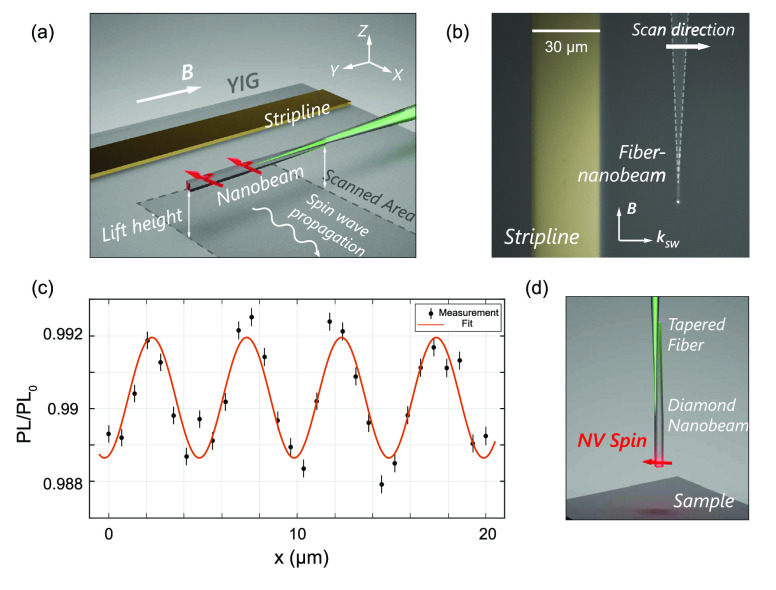
Proof-of-principle scanning NV magnetometry with a fiber-coupled diamond nanobeam. (a) Experimental geometry: A fiber-coupled NV nanobeam is used to image spin waves in a 250 nm thick film of yttrium-iron garnet. The YIG–nanobeam distance is ∼5 μm. The beam is scanned perpendicularly to the beam axis. The spin waves are excited by a microwave current in a gold stripline (3 mm × 30 μm). An auxiliary wire drawn across the chip (not shown) provides a reference field that interferes with the spin-wave stray field, creating a standing-wave pattern in the microwave magnetic-field amplitude.^25^ A static magnetic field *B* is applied along the beam direction. (b) Microscope image of the experimental geometry. (c) Scanning-nanobeam imaging of a spin wave. The NV ESR contrast PL/PL_0_ is measured by switching on and off the microwave drive at the NV ESR frequency. The error bars are estimated from assuming shot noise of the NV photoluminescence during the measurement time (SI). A sinusoidal fit (orange) gives the measured wavelength λ = 5.0(1) μm. (d) Envisioned single-NV scanning diamond nanobeam for high-resolution 2D imaging.

To demonstrate the imaging capability of our fiber-coupled nanobeam sensor, we use it to image spin waves, the wave-like excitations of spins in a magnetic material,^39^ in a ∼250 nm thick film of YIG.^40^ We excite the spin wave by sending a microwave current through a gold stripline on the YIG (Figure 4a) under a static external magnetic field *B* = 22 mT. The spin wave generates a microwave magnetic stray field that drives the NV spins when its frequency matches the NV ESR frequency. To create a spatial standing-wave pattern in the microwave field that we can image via the NV ESR contrast (SI), we apply an additional, spatially homogeneous reference field of the same microwave frequency using a wire above the chip.^11,25^ We scan the beam parallel to the sample surface and perpendicularly to the beam axis (Figure 4a,b), and measure the NV ESR contrast by switching on and off the microwave drive at the ESR frequency *f* = 2.439 GHz. The result shown in Figure 4c images the spin-wavefront in 1D with a resolution limited by the beam width and beam-sample distance. The observed wavelength of λ = 5.0 μm corresponds reasonably well with the 6 μm expected from the spin-wave dispersion (SI), given the uncertainty in the angle of the applied magnetic field.

## Conclusion

To conclude, we demonstrated a new fiber-based approach for scanning NV magnetometry measurements. Using quasi-isotropic etching, we nanofabricate diamond nanobeams out of single-crystal bulk diamond and couple them to tapered optical fibers. We read out ensemble NV signals through the fiber–nanobeam coupling with an estimated efficiency of 8.6(4)% at the coupling interface. As a demonstration, we show that our device can function as a scanning sensor to measure in 1D the planar spin wave in YIG.

A remaining challenge lies in increasing the control over the angle and position when attaching the nanobeam to the fiber. While we found that we can consistently break off the beams and attach them to a fiber, their relative position after the breaking process is not entirely under control due to the abrupt motion of the fiber-nanobeam when the tether breaks. We expect that reducing the tether width further, or reducing the nanobeam surface roughness via improved etching or ion-based polishing,^37^ could improve the coupling efficiency. Additionally, transporting the fiber-nanobeam probe is challenging due to vibration and static electricity that cause the nanobeam to detach. We found that the nanobeams stay attached to the fiber as long as the devices are kept fixed in the setup. For instance, the nanobeam used for scanning in Figure 4 remained attached to the fiber for more than two months. A still outstanding challenge is the development of a method for dismounting the nanobeam–fiber assembly and transporting it to a different setup. Envisioned ways to address this challenge include the design of dedicated fiber holders, followed by evaporation of dielectric materials onto the assembly.

Further steps toward 2D magnetic imaging (Figure 4d) include deterministically placing NV centers at the end of the nanobeams by, e.g., prelocalizing NV centers^41^ or deterministic implantation.^42^ With above-mentioned efforts, our work holds potential for implementation in low-temperature setups with reduced heat load and easier alignment, opening another possibility for imaging weak magnetic effects at low temperature, e.g., currents in quantum Hall devices^43^ and Josephson junctions.^44^

## Methods

### Magnetometry with NV Centers

The NV center is a spin-1 system. The ground state of an NV center splits into three spin substates *m*_S_ = 0, ±1, and the microwave-driven transition between *m*_S_ = 0 and *m*_S_ = ±1 states can be detected via the photoluminescence intensity under nonresonant green laser excitation: Once the frequency of applied microwave matches the *m*_S_ = 0 → ±1 transition frequencies (ESR frequencies), the photoluminescence emission of the NV center will decrease due to the higher nonradiative decay rate of the *m*_S_ = ±1 states. Applying an external magnetic field lifts the degeneracy of the *m*_S_ = ±1 states, allowing magnetic field measurement through measuring the ESR frequencies. More detailed information on the working principle of NV centers can be found in refs (1−4).

### Device Fabrication

We fabricate the diamond nanobeams on single crystal CVD diamond (element-six DNV-B14) with ensemble NV centers generated during growth. Before fabrication, we mechanically polish the diamond surface down to *R*_*a*_ ∼ 2 nm (Almax EasyLab) and clean the diamond chip with fuming nitric acid (HNO_3_).

To fabricate the beams, we first deposit a 200 nm layer of Si_3_N_4_ on the surface with PECVD (20 sccm SiH_4_/20 sccm NH_3_/980 sccm N_2_, deposited at 300 °C, Oxford Instruments Plasmalab 80 Plus) as the hard mask. We then spin-coat a ∼400 nm layer of e-beam resist (AR-P 6200-13) and a ∼30 nm conductive layer of Elektra-92 on top to write the pattern with e-beam lithography (Raith EBPG5200). We transfer the e-beam pattern from the resist to the SiN hard mask by an anisotropic ICP-RIE etch with CHF_3_/O_2_ (60 sccm/6 sccm, 50 W RF and 500 W ICP at 20 °C, AMS 100 I-speeder). We then remove the resist with dimethylformamide (DMF) and subsequent Piranha cleaning (96% H_2_SO_4_ and 31% H_2_O_2_, 3:1 mixed at 80 °C). An ICP-RIE etch with O_2_ (50 sccm, 90 W RF and 1100 W ICP at 20 °C, Oxford Instruments Plasmalab 100) transfers the pattern onto the diamond.

To protect the sidewalls of the structure during the subsequent undercut etch, we deposit ∼20 nm of Al_2_O_3_ with ALD (280 cycles, at 105 °C, Oxford Instruments FlexAL) and remove the Al_2_O_3_ on the topside with another ICP-RIE with BCl_3_/Cl_2_ (45 sccm/5 sccm, 10 W RF and 600 W ICP at 20 °C, Oxford Instruments Plasmalab 100). We do the final undercut of the beams with quasi-isotropic O_2_ ICP-RIE at 65 °C (50 sccm, 0 W RF, and 2500 W ICP, Oxford Instruments Plasmalab 100). For the sample discussed in the main text, completely undercutting the ∼500 nm wide nanobeams took 12 h. The etch rate of our quasi-isotropic etching process is mainly limited by the maximum etching temperature of our system, and varies over different design, sample and etcher condition. All the masks are eventually cleaned with 40% hydrofluoric acid (HF, 10 min). Further details of the relevant recipe parameters can be found in ref (22).

The net cleanroom processing time for one complete fabrication flow is around 20 h, which we spread over 4–5 working days in practice. For each fabrication flow, more than 200 nanobeams can be fabricated on a 2 × 2 mm^2^ diamond chip.

We fabricate the tapered fibers by wet etching commercial optical fibers (S630-HP) with 40% HF. We dip one end of the fiber into the acid and pull it out at constant speed using a motorized translation stage (Thorlabs MTS25-Z8). The tapering angle can thus be controlled by tuning the pulling speed.^15^

## Data Availability

All data plotted in the figures of this work are available at zenodo.org with identifier DOI: 10.5281/zenodo.7561825. Additional data related to this paper are available upon request.

## Supporting Information Available

Estimation of coupling efficiency using the absorption cross section method; Additional characterization of different fiber-nanobeam devices; Explanation on the spin wave dispersion in YIG and the principles of using NV centers to image propagating spin waves; Uncertainty evaluation of spin wave imaging (PDF)

## References

[ref1] Schirhagl R.; Chang K.; Loretz M.; Degen C. L. Nitrogen-vacancy centers in diamond: Nanoscale sensors for physics and biology. Annu. Rev. Phys. Chem. 2014, 65, 83–105. 10.1146/annurev-physchem-040513-103659. 24274702

[ref2] Rondin L.; Tetienne J. P.; Hingant T.; Roch J. F.; Maletinsky P.; Jacques V. Magnetometry with nitrogen-vacancy defects in diamond. Rep. Prog. Phys. 2014, 77, 056503 10.1088/0034-4885/77/5/056503. 24801494

[ref3] Doherty M. W.; Manson N. B.; Delaney P.; Jelezko F.; Wrachtrup J.; Hollenberg L. C. The nitrogen-vacancy colour centre in diamond. Phys. Rep. 2013, 528, 1–45. 10.1016/j.physrep.2013.02.001.

[ref4] Casola F.; Van Der Sar T.; Yacoby A. Probing condensed matter physics with magnetometry based on nitrogen-vacancy centres in diamond. Nature Reviews Materials 2018, 3, 1–13. 10.1038/natrevmats.2017.88.

[ref5] Mochalin V. N.; Shenderova O.; Ho D.; Gogotsi Y. The properties and applications of nanodiamonds. Nat. Nanotechnol. 2012, 7, 11–23. 10.1038/nnano.2011.209. 22179567

[ref6] Le Sage D.; Arai K.; Glenn D. R.; Devience S. J.; Pham L. M.; Rahn-Lee L.; Lukin M. D.; Yacoby A.; Komeili A.; Walsworth R. L. Optical magnetic imaging of living cells. Nature 2013, 496, 486–489. 10.1038/nature12072. 23619694 PMC3641584

[ref7] Barry J. F.; Turner M. J.; Schloss J. M.; Glenn D. R.; Song Y.; Lukin M. D.; Park H.; Walsworth R. L. Optical magnetic detection of single-neuron action potentials using quantum defects in diamond. Proc. Natl. Acad. Sci. U.S.A. 2016, 113, 14133–14138. 10.1073/pnas.1601513113. 27911765 PMC5150388

[ref8] Glenn D. R.; Fu R. R.; Kehayias P.; Le Sage D.; Lima E. A.; Weiss B. P.; Walsworth R. L. Micrometer-scale magnetic imaging of geological samples using a quantum diamond microscope. Geochemistry, Geophysics, Geosystems 2017, 18, 3254–3267. 10.1002/2017GC006946.

[ref9] Maletinsky P.; Hong S.; Grinolds M. S.; Hausmann B.; Lukin M. D.; Walsworth R. L.; Loncar M.; Yacoby A. A robust scanning diamond sensor for nanoscale imaging with single nitrogen-vacancy centres. Nat. Nanotechnol. 2012, 7, 320–324. 10.1038/nnano.2012.50. 22504708

[ref10] Gross I.; et al. Real-space imaging of non-collinear antiferromagnetic order with a single-spin magnetometer. Nature 2017, 549, 252–256. 10.1038/nature23656. 28905889

[ref11] Simon B. G.; Kurdi S.; La H.; Bertelli I.; Carmiggelt J. J.; Ruf M.; De Jong N.; Van Den Berg H.; Katan A. J.; Van Der Sar T. Directional Excitation of a High-Density Magnon Gas Using Coherently Driven Spin Waves. Nano Lett. 2021, 21, 8213–8219. 10.1021/acs.nanolett.1c02654. 34597058 PMC8517981

[ref12] Fedotov I. V.; Doronina-Amitonova L. V.; Sidorov-Biryukov D. A.; Safronov N. A.; Blakley S.; Levchenko A. O.; Zibrov S. A.; Fedotov A. B.; Kilin S. Y.; Scully M. O.; Velichansky V. L.; Zheltikov A. M. Fiber-optic magnetic-field imaging. Opt. Lett. 2014, 39, 6954 10.1364/OL.39.006954. 25503039

[ref13] Chatzidrosos G.; Rebeirro J. S.; Zheng H.; Omar M.; Brenneis A.; Stürner F. M.; Fuchs T.; Buck T.; Rölver R.; Schneemann T.; Blümler P.; Budker D.; Wickenbrock A. Fiberized Diamond-Based Vector Magnetometers. Frontiers in Photonics 2021, 2, 4 10.3389/fphot.2021.732748.

[ref14] Tiecke T. G.; Nayak K. P.; Thompson J. D.; Peyronel T.; de Leon N. P.; Vuletić V.; Lukin M. D. Efficient fiber-optical interface for nanophotonic devices. Optica 2015, 2, 70 10.1364/OPTICA.2.000070.

[ref15] Burek M. J.; Meuwly C.; Evans R. E.; Bhaskar M. K.; Sipahigil A.; Meesala S.; MacHielse B.; Sukachev D. D.; Nguyen C. T.; Pacheco J. L.; Bielejec E.; Lukin M. D.; Lončar M. Fiber-coupled diamond quantum nanophotonic interface. Physical Review Applied 2017, 8, 024026 10.1103/PhysRevApplied.8.024026.

[ref16] Gröblacher S.; Hill J. T.; Safavi-Naeini A. H.; Chan J.; Painter O. Highly efficient coupling from an optical fiber to a nanoscale silicon optomechanical cavity. Appl. Phys. Lett. 2013, 103, 181104 10.1063/1.4826924.

[ref17] Burek M. J.; Chu Y.; Liddy M. S.; Patel P.; Rochman J.; Meesala S.; Hong W.; Quan Q.; Lukin M. D.; Loncar M. High quality-factor optical nanocavities in bulk single-crystal diamond. Nat. Commun. 2014, 5, 5718 10.1038/ncomms6718. 25511421

[ref18] Mouradian S.; Wan N. H.; Schröder T.; Englund D. Rectangular photonic crystal nanobeam cavities in bulk diamond. Appl. Phys. Lett. 2017, 111, 021103 10.1063/1.4992118.

[ref19] Bradac C.; Gao W.; Forneris J.; Trusheim M. E.; Aharonovich I. Quantum nanophotonics with group IV defects in diamond. Nat. Commun. 2019, 10, 5625 10.1038/s41467-019-13332-w. 31819050 PMC6901484

[ref20] Khanaliloo B.; Mitchell M.; Hryciw A. C.; Barclay P. E. High-Q/V Monolithic Diamond Microdisks Fabricated with Quasi-isotropic Etching. Nano Lett. 2015, 15, 5131–5136. 10.1021/acs.nanolett.5b01346. 26134379

[ref21] Wan N. H.; Lu T. J.; Chen K. C.; Walsh M. P.; Trusheim M. E.; De Santis L.; Bersin E. A.; Harris I. B.; Mouradian S. L.; Christen I. R.; Bielejec E. S.; Englund D. Large-scale integration of artificial atoms in hybrid photonic circuits. Nature 2020, 583, 226–231. 10.1038/s41586-020-2441-3. 32641812

[ref22] Ruf M. T. Cavity-enhanced quantum network nodes in diamond. Ph.D. thesis, TU Delft, 2021.

[ref23] Thompson J. D.; Tiecke T. G.; de Leon N. P.; Feist J.; Akimov A. V.; Gullans M.; Zibrov A. S.; Vuletić V.; Lukin M. D. Coupling a Single Trapped Atom to a Nanoscale Optical Cavity. Science 2013, 340, 1202–1205. 10.1126/science.1237125. 23618764

[ref24] Magrini L.; Norte R. A.; Riedinger R.; Marinković I.; Grass D.; Delić U.; Gröblacher S.; Hong S.; Aspelmeyer M. Near-field coupling of a levitated nanoparticle to a photonic crystal cavity. Optica 2018, 5, 1597 10.1364/OPTICA.5.001597.

[ref25] Bertelli I.; Carmiggelt J. J.; Yu T.; Simon B. G.; Pothoven C. C.; Bauer G. E. W.; Blanter Y. M.; Aarts J.; Van Der Sar T. Magnetic resonance imaging of spin-wave transport and interference in a magnetic insulator. Science Advances 2020, 6, 3556–3567. 10.1126/sciadv.abd3556. PMC7673737 33177096

[ref26] Marinković I.; Drimmer M.; Hensen B.; Gröblacher S. Hybrid integration of silicon photonic devices on lithium niobate for optomechanical wavelength conversion. Nano Lett. 2021, 21, 529–535. 10.1021/acs.nanolett.0c03980. 33393311 PMC7809686

[ref27] Ruoff A. L. On the yield strength of diamond. J. Appl. Phys. 1979, 50, 3354–3356. 10.1063/1.326378.

[ref28] Banerjee A.; Bernoulli D.; Zhang H.; Yuen M. F.; Liu J.; Dong J.; Ding F.; Lu J.; Dao M.; Zhang W.; Lu Y.; Suresh S. Ultralarge elastic deformation of nanoscale diamond. Science 2018, 360, 300–302. 10.1126/science.aar4165. 29674589

[ref29] Element Six, DNV Series Datasheet. 2021; https://e6cvd.com/uk/material/single-crystalline/dnv-b14-203-0mmx3-0mm-0-5mm.html, accessed 2022–12–18.

[ref30] Dréau A.; Lesik M.; Rondin L.; Spinicelli P.; Arcizet O.; Roch J. F.; Jacques V. Avoiding power broadening in optically detected magnetic resonance of single NV defects for enhanced dc magnetic field sensitivity. Physical Review B - Condensed Matter and Materials Physics 2011, 84, 195204 10.1103/PhysRevB.84.195204.

[ref31] Manson N. B.; Harrison J. P.; Sellars M. J. Nitrogen-vacancy center in diamond: Model of the electronic structure and associated dynamics. Physical Review B - Condensed Matter and Materials Physics 2006, 74, 104303 10.1103/PhysRevB.74.104303.

[ref32] Aslam N.; Waldherr G.; Neumann P.; Jelezko F.; Wrachtrup J. Photo-induced ionization dynamics of the nitrogen vacancy defect in diamond investigated by single-shot charge state detection. New J. Phys. 2013, 15, 013064 10.1088/1367-2630/15/1/013064.

[ref33] Siyushev P.; Nesladek M.; Bourgeois E.; Gulka M.; Hruby J.; Yamamoto T.; Trupke M.; Teraji T.; Isoya J.; Jelezko F. Photoelectrical imaging and coherent spin-state readout of single nitrogen-vacancy centers in diamond. Science 2019, 363, 728–731. 10.1126/science.aav2789. 30765564

[ref34] Wee T. L.; Tzeng Y. K.; Han C. C.; Chang H. C.; Fann W.; Hsu J. H.; Chen K. M.; Yu E. C. Two-photon excited fluorescence of nitrogen-vacancy centers in proton-irradiated type Ib diamond. J. Phys. Chem. A 2007, 111, 9379–9386. 10.1021/jp073938o. 17705460

[ref35] Robledo L.; Bernien H.; Van Der Sar T.; Hanson R. Spin dynamics in the optical cycle of single nitrogen-vacancy centres in diamond. New J. Phys. 2011, 13, 025013 10.1088/1367-2630/13/2/025013.

[ref36] Beveratos A.; Brouri R.; Gacoin T.; Poizat J.-P.; Grangier P. Nonclassical radiation from diamond nanocrystals. Phys. Rev. A 2001, 64, 061802 10.1103/PhysRevA.64.061802.

[ref37] Mi S.; Toros A.; Graziosi T.; Quack N. Non-contact polishing of single crystal diamond by ion beam etching. Diamond Relat. Mater. 2019, 92, 248–252. 10.1016/j.diamond.2019.01.007.

[ref38] Fujii T.; Taguchi Y.; Saiki T.; Nagasaka Y. A fusion-spliced near-field optical fiber probe using photonic crystal fiber for nanoscale thermometry based on fluorescence-lifetime measurement of quantum dots. Sensors 2011, 11, 8358–8369. 10.3390/s110908358. 22164080 PMC3231516

[ref39] Chumak A. V.; Vasyuchka V. I.; Serga A. A.; Hillebrands B. Magnon spintronics. Nat. Phys. 2015, 11, 453–461. 10.1038/nphys3347.

[ref40] Serga A. A.; Chumak A. V.; Hillebrands B. YIG magnonics. J. Phys. D: Appl. Phys. 2010, 43, 264002 10.1088/0022-3727/43/26/264002.

[ref41] Wan N. H.; Shields B. J.; Kim D.; Mouradian S.; Lienhard B.; Walsh M.; Bakhru H.; Schröder T.; Englund D. Efficient Extraction of Light from a Nitrogen-Vacancy Center in a Diamond Parabolic Reflector. Nano Lett. 2018, 18, 2787–2793. 10.1021/acs.nanolett.7b04684. 29601205

[ref42] Schukraft M.; Zheng J.; Schröder T.; Mouradian S. L.; Walsh M.; Trusheim M. E.; Bakhru H.; Englund D. R. Precision nanoimplantation of nitrogen vacancy centers into diamond photonic crystal cavities and waveguides. APL Photonics 2016, 1, 020801 10.1063/1.4948746.

[ref43] Uri A.; Kim Y.; Bagani K.; Lewandowski C. K.; Grover S.; Auerbach N.; Lachman E. O.; Myasoedov Y.; Taniguchi T.; Watanabe K.; Smet J.; Zeldov E. Nanoscale imaging of equilibrium quantum Hall edge currents and of the magnetic monopole response in graphene. Nat. Phys. 2020, 16, 164–170. 10.1038/s41567-019-0713-3.

[ref44] Roditchev D.; Brun C.; Serrier-Garcia L.; Cuevas J. C.; Bessa V. H. L.; Miloševic M. V.; Debontridder F.; Stolyarov V.; Cren T. Direct observation of Josephson vortex cores. Nat. Phys. 2015, 11, 332–337. 10.1038/nphys3240.

