# Supplementary material for: A Fiber-Coupled Scanning Magnetometer with Nitrogen-Vacancy Spins in a Diamond Nanobeam
Source: ACS Photonics. 2023 May 25;10(6):1859–65. doi: 10.1021/acsphotonics.3c00259 (PMC10288530; doi:10.1021/acsphotonics.3c00259)
Supplement: Supplementary file 1 — ph3c00259_si_001.pdf [file ph3c00259_si_001.pdf]

Supporting Information for "A Fiber-Coupled Scanning Magnetometer with Nitrogen-Vacancy Spins in a Diamond Nanobeam"

Yufan Li,[†,‡] Fabian A. Gerritsma,[†] Samer Kurdi,[†] Nina Codreanu,[¶] Simon Gröblacher,[†] Ronald Hanson,[¶] Richard Norte,[†,‡] and Toeno van der Sar[*,†]

†*Department of Quantum Nanoscience, Kavli Institute of Nanoscience, Delft University of Technology, Delft 2628CJ, The Netherlands*

‡*Department of Precision and Microsystems Engineering, Faculty of Mechanical, Maritime and Materials Engineering, Delft University of Technology, Delft 2628CD, The Netherlands*

¶*QuTech and Kavli Institute of Nanoscience, Delft University of Technology, Delft 2628CJ, The Netherlands*

E-mail: t.vandersar@tudelft.nl

Estimating the Collection Efficiency through Absorption Cross Section

Consider a single NV center inside a diamond nanobeam. If the area of the optical mode cross section inside the beam is approximately the cross section of the nanobeam itself A_{beam}, then the probability for an NV center to absorb a single photon and trigger an excitation will be

$$\mathcal{P} = \frac{\sigma}{A_{\mathrm{beam}}} \tag{1}$$

where $\sigma = 3.1(8) \times 10^{-21}\,\mathrm{m}^2$ is the absorption cross section of the NV center.[1] Therefore when a total of N_{ph} photons enter a beam containing N_{NV} NVs, the total number of excitations (thus total number of emitted photons) will be

$$N_{\mathrm{PL}} = \frac{N_{\mathrm{ph}} N_{\mathrm{NV}} \sigma}{A_{\mathrm{beam}}}. \tag{2}$$

Note that the effect of saturation discussed in the main text is not taken into account here, thus the optical power needs to stay well below saturation for this equation to hold.

Now consider a power of P being sent into the fiber with a fiber-coupling efficiency of η_{f}. The rate of excitation photons that eventually end up in the beam mode will then be

$$\Gamma_{\mathrm{exc}} = \frac{P}{\hbar\omega} \eta_{\mathrm{f}} \eta_{\mathrm{nf}} \tag{3}$$

where η_{nf} is the coupling efficiency at the fiber-nanobeam interface. Thus the photoluminescence rate will be

$$\Gamma_{\mathrm{PL}} = \frac{P}{\hbar\omega} \frac{N_{\mathrm{NV}} \sigma}{A_{\mathrm{beam}}} \eta_{\mathrm{f}} \eta_{\mathrm{nf}} \tag{4}$$

and the measured photon rate is (assuming coupling efficiency at the fiber-nanobeam inter-

face is equal for both directions)

$$\Gamma_{\text{meas}} = \frac{P}{\hbar\omega}\frac{N_{\text{NV}}\sigma}{A_{\text{beam}}}\eta_{\text{f}}\eta^2\eta_{\text{D}} \tag{5}$$

where η_{D} is the fraction of photons exiting the fiber that are eventually detected by the APD.

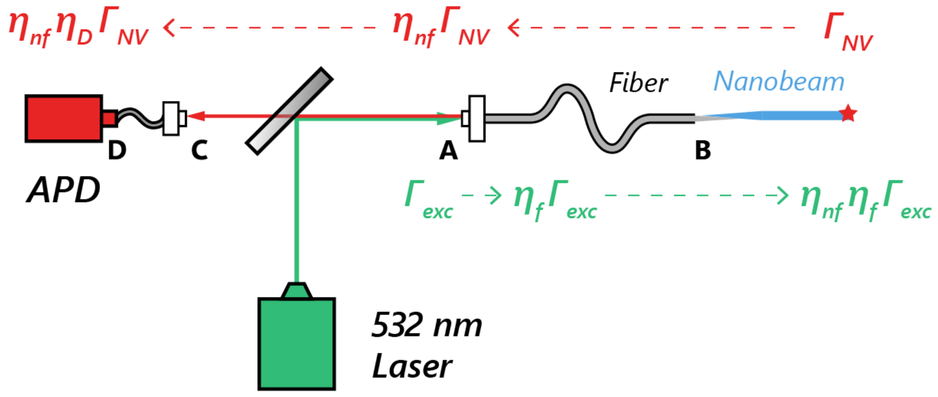

Figure S1: Overview of the efficiencies involved in our measurement. Γ_{exc}: Photon rate of excitation laser. η_{f}: Efficiency of free-space excitation laser coupling into the fiber. η_{nf}: Efficiency of adiabatic light coupling at the fiber-nanobeam interface. Γ_{NV}: Photoluminescence rate of NV centers inside the nanobeam. η_{D}: fraction of total photons exiting the fiber that are eventually detected by the APD, consisting of losses in free space optics (e.g. on mirrors and filters), fiber coupling efficiency at the APD input and the detection efficiency of the APD.

Therefore from eq. (5) one can estimate η_{nf} from P and $\mathcal{R}_{\text{meas}}$, both of which are measured experimentally. For the device mentioned in the main text, we measured $\Gamma_{\text{meas}} = 1.08 \times 10^6\,\text{s}^{-1}$ at $P = 30\,\text{nW}$. For the efficiencies, fig. S1 shows a simplified overview of the measurement setup and the efficiencies involved. To experimentally determine η_{f}, we connect a non-tapered fiber (same model as the tapered one) to the fiber coupler (A), send in a green

laser with power P_{in} measured in front of the coupler, measure the power at the output (B) of the fiber P_{out} and determine $\eta_{\text{f}} = P_{\text{out}}/P_{\text{in}} \approx 0.35$. For η_{D}, we send in a red laser from B, measure the power at fiber output (A) and the APD fiber input (C and D), and determine $\eta_{\text{D}} \approx 0.035$ (including the efficiency of the free-space optical components $\eta_{\text{A}\to\text{C}} \approx 0.5$, APD fiber coupling efficiency $\eta_{\text{C}\to\text{D}} \approx 0.1$ and APD detection efficiency $\eta_{\text{APD}} = 0.7$). Substituting all the numbers into eq. (5), we can calculate the coupling efficiency at the interface

$$\eta_{\text{nf}} = 14(2)\%. \tag{6}$$

Additional Characterization of Fiber-Nanobeam Devices

As stated in the main text, the coupling efficiency of our device is sensitive to the alignment of the tapered fiber and the diamond nanobeam. The precise alignment is hindered by the abrupt motion of the fiber-nanobeam when the tether breaks, thus inducing the variation of coupling efficiency across different devices. Aside from the device demonstrated in the main text, in fig. S2 we show ESR measurements on four different nanobeams from the same fabrication batch and coupled to the same tapered fiber, along with their estimated coupling efficiency from the absorption cross section method.

The effect of fiber-nanobeam alignment on the coupling efficiency is further studied by measuring ESR of the nanobeams without breaking them off the bulk. This allows optimizing the relative position of the fiber tip and the nanobeam by monitoring the photon count while adjusting the positioning of the fiber tip. Such a measurement is performed on a 30 µm beam (maximum cross section $0.5 \times 0.5\,\mu\text{m}^2$, tapered down to $\sim 0.1 \times 0.5\,\mu\text{m}^2$ over 27 µm length) attached to the bulk, with optimized photon counts (fig. S3). This measurement yields $\eta_{\text{nf}} = 19(3)\%$.

We also measured the ESR spectrum on the bulk of the same diamond through free space confocal illumination and collection, shown in fig. S4. Here we also observe the splitting of

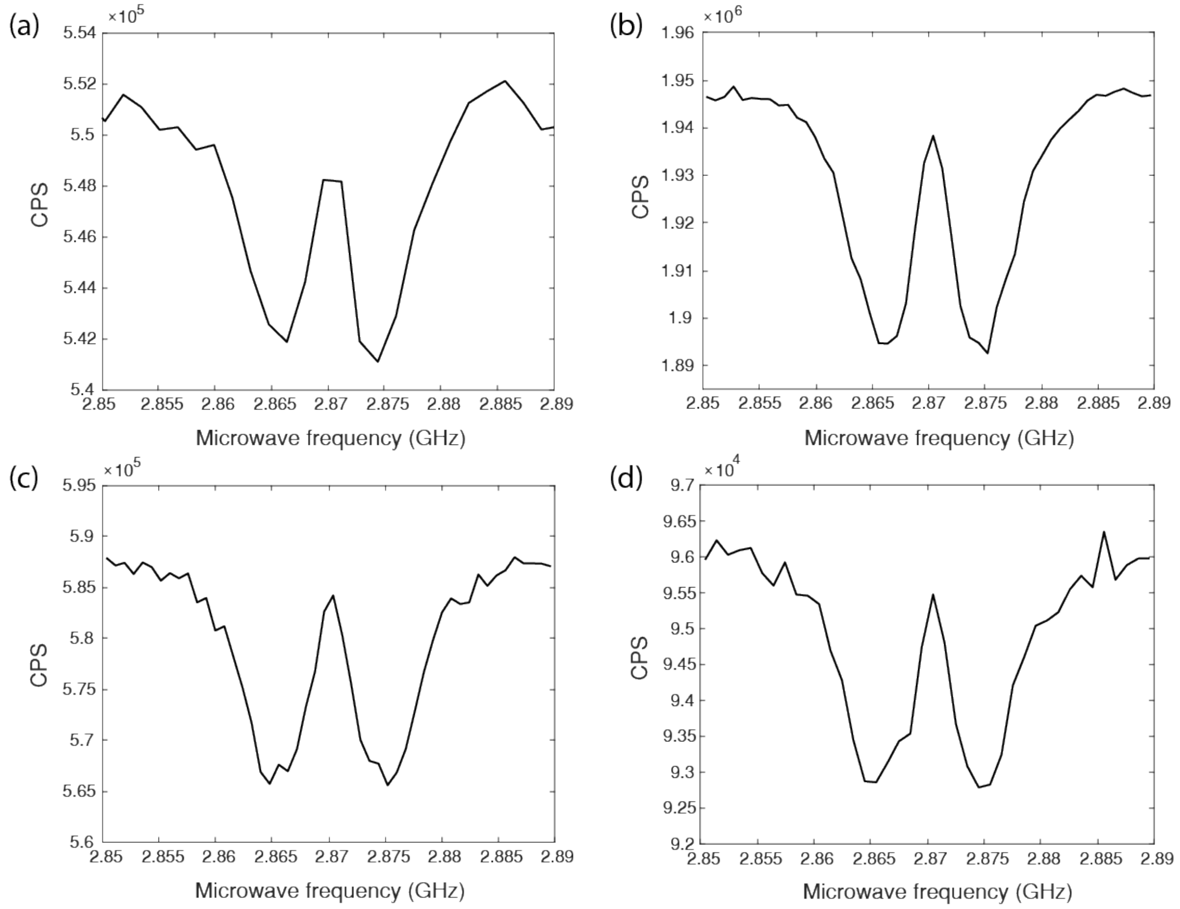

Figure S2: ESR spectrum of four different nanobeam devices, measured at excitation power of (a) 35 nW; (b) 30 nW; (c) 25 nW; (d) 40 nW plus ND 1.3 filtering. Using absorption cross section method explained in the previous section, coupling efficiencies on the fiber-nanobeam interfaces are estimated to be (a) 9(1)% (b) 19(3)% (c) 11(2)% (d) 16(2)%.

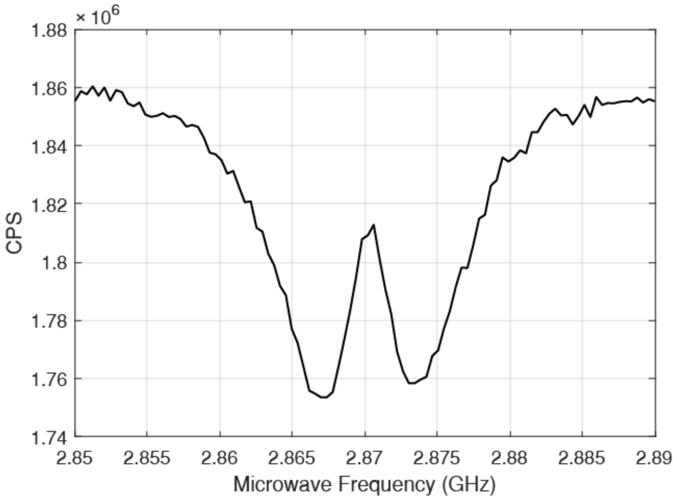

Figure S3: ESR spectrum of a nanobeam that remains attached to the bulk of the diamond, measured at excitation power of 31 nW. This beam has a total length of 30 µm instead of 40 µm for the beams measured in the main text and in fig. S2, and for this measurement we have a different $\eta_D = 0.042$ due to switching of the collection fiber in front of the APD. Efficiency estimation on the fiber-nanobeam interface is estimated to be 19(3)%.

the ESR dips in the absence of external field, indicating that this is intrinsic to the specific diamond we use rather than the nanobeam geometry.

Figure S5 shows the spectrum of the photoluminescence measured from our device. A shift towards the NV^0 spectrum is visible for excitation power above around 1 µW, indicating minimal NV^0 contribution in our low-power ESR measurements.

Imaging the Spin Waves in YIG with NV centers

Spin waves can be excited in the YIG thin film with the AC magnetic field we apply through the stripline. To model the spin dynamics, we consider our YIG layer as an infinite film parallel to the xy plane, infinite in x and y, and with a thickness t in z direction. We can calculate the dispersion of the spin waves using the Landau-Lifshitz-Gilbert (LLG) equation

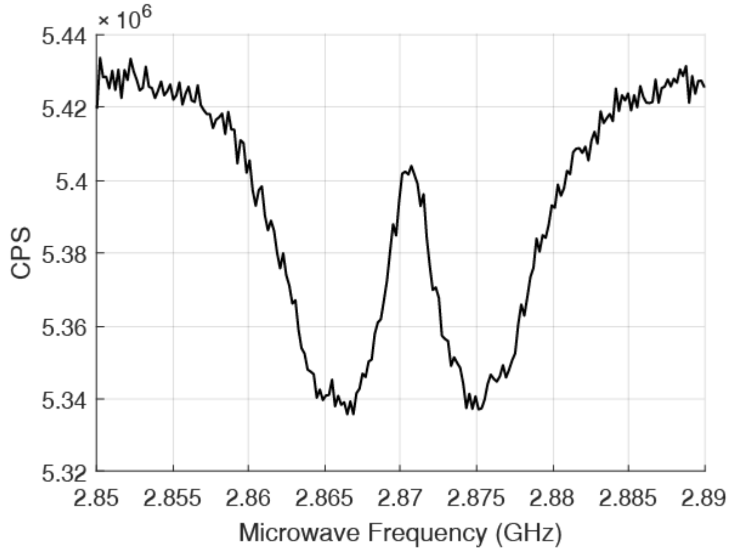

Figure S4: ESR spectrum measured on the bulk of the same diamond. Free space laser excitation of 350 nW is focused on the surface of the diamond via a 50× microscope objective in a confocal microscope, and NV photoluminescence is also collected using the same objective.

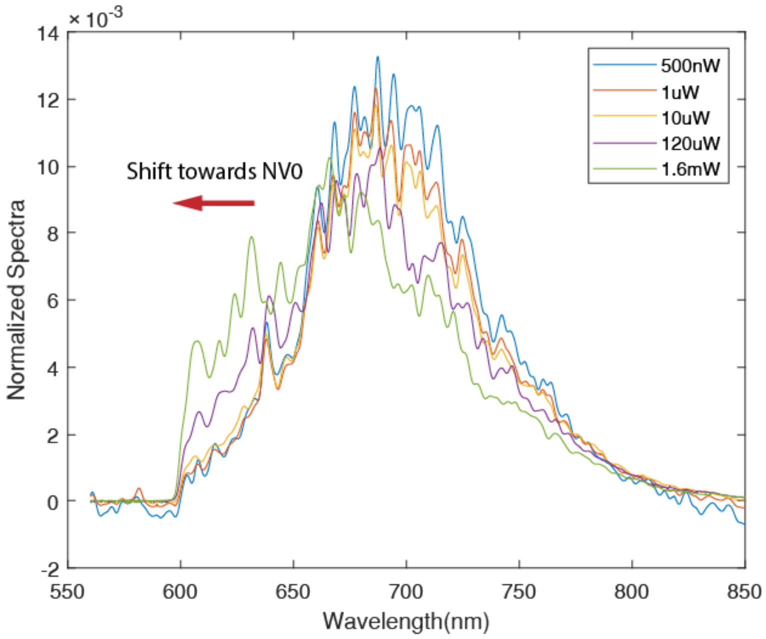

Figure S5: Spectrum of detected photoluminescence at different excitation power, taken after 600 nm long-pass filtering. The oscillation on top of the curves can be a result from the optical interference in the nanobeam or within the spectrometer (Thorlabs CCS100).

which governs the dynamics of magnetization:

$$\dot{\mathbf{m}} = -\gamma \mathbf{m} \times (\mathbf{B}_{\text{eff}} + \mathbf{B}_{\text{AC}}) - \alpha \dot{\mathbf{m}} \times \mathbf{m} \tag{7}$$

in which $\mathbf{m}(\mathbf{r})$ is the unit vector in the direction of the magnetization $\mathbf{M}(\mathbf{r}) = M_s \mathbf{m}(\mathbf{r})$ (with M_s being the saturation magnetization), $\mathbf{B}_{\text{AC}}$ is the AC driving field, γ is the gyromagtic ratio and α is the Gilbert damping. The effective field $\mathbf{B}_{\text{eff}}$ takes into account the static external magnetic field $\mathbf{B}_0$, the demagnetizing field $\mathbf{B}_d$ and the exchange interaction.

Since spin waves are propagating waves of spin precession around the magnetization axis in equilibrium, we are interested in the dynamics of the *transverse* magnetization

$$\mathbf{m}_{\perp} = \chi \mathbf{B}_{\text{AC},\perp} \tag{8}$$

where $\mathbf{m}_{\perp} = m_{x'} \hat{\mathbf{x}}' + m_{y'} \hat{\mathbf{y}}'$ is the transverse components of the magnetization vector, with the magnetic frame (x', y', z') defined such that $\mathbf{m}(\mathbf{r}) = \hat{\mathbf{z}}'$ in equilibrium. $\mathbf{B}_{\text{AC},\perp} = \mathbf{B}_{\text{AC,x}'} \hat{\mathbf{x}}' + \mathbf{B}_{\text{AC,y}'} \hat{\mathbf{y}}'$ is the transverse drive field in the magnetic frame, and χ is the *transverse susceptibility.* Following the formalism by Rustagi et al.,[2] we can find χ by solving the LLG equation in k-space, and determine the spin wave dispersion $\omega_{\text{sw}}(\mathbf{k})$ by finding the singularity of χ, which eventually gives us:

$$\omega_{\text{sw}}(\mathbf{k}) = \sqrt{\omega_2 \omega_3 - \omega_1^2}, \tag{9}$$

where $\mathbf{k} = (k_x, k_y)$ is the in-plane wave vector. Parameters $\omega_1 \sim \omega_3$ are in general functions of the angle between the z and z' axes, the direction of $\mathbf{k}$, effective static field and the film thickness.[2] In our measurements, we apply the external magnetic field in-plane, and we excite and detect spin waves in the Damon-Eshbach (DE) regime where the spin wave wave vector $\mathbf{k}$ is perpendicular to the external magnetic field $\mathbf{B}_0$. Under these conditions, $\omega_1 \sim \omega_3$

are reduced to:

$$\omega_1 = 0; \tag{10}$$

$$\omega_2 = \omega_B + \omega_D k^2 + \omega_M(1 - f); \tag{11}$$

$$\omega_3 = \omega_B + \omega_D k^2 + \omega_M f. \tag{12}$$

where we defined $\omega_B = \gamma B_0$, $\omega_D = \gamma D/M_s$ with D being the spin stiffness that characterizes the exchange interaction between spins, $\omega_M = \gamma\mu_0 M_s$, $f = 1 - (1 - e^{-kt})/kt$ with t being the film thickness.

The spin waves generate an AC magnetic field at the spin wave frequency ω_{sw}, and the amplitude of the field varies in space according to the wave number k. In our system we use a stripline to excite spin waves traveling in x-direction with a planar wave front, which can be written as

$$B_{\mathrm{sw}}(x,t) = B_{\mathrm{sw},0} e^{i(kx+\omega_{\mathrm{sw}}t)}. \tag{13}$$

This field can then drive the NV-ESR transitions when the spin wave frequency matches the ESR frequency. In order to image the spatial variation of B_{sw}, we apply a homogeneous reference field with the same frequency $B_{\mathrm{ref}}(t) = B_{\mathrm{ref},0} e^{i\omega_{\mathrm{sw}}t}$ through a bonding wire located above the sample. This creates a spatial variation in the field amplitude at the spin wave wavelength:

$$|B_{\mathrm{sw}}(x,t) + B_{\mathrm{ref}}(t)|^2 = B_{\mathrm{sw},0}^2 + B_{\mathrm{ref},0}^2 + 2B_{\mathrm{sw},0} B_{\mathrm{ref},0} \cos kx. \tag{14}$$

This results in a spatially varying Rabi frequency of the NV-ESR transition

$$\Omega_R(x) = \frac{\gamma}{\sqrt{2}} |B_{\mathrm{sw}}(x,t) + B_{\mathrm{ref}}(t)| \tag{15}$$

which directly determines the NV-ESR contrast $\mathcal{C} = (\mathrm{PL} - \mathrm{PL}_0)/\mathrm{PL}_0$ (defined in the main

text) through the relation[3]

$$\mathcal{C}(x) \propto \frac{\Omega_R^2(x)}{\Omega_R^2(x) + \Delta} \tag{16}$$

where the parameter Δ is determined by the optical excitation power of NV centers, therefore remains constant in our measurement scheme. As a result, the measured ESR contrast along the spin wave propagation direction has the same periodicity as the spin wave itself, allowing the imaging of spin wave through measuring ESR contrast.

Furthermore, from the ESR frequency one can determine both the detected spin wave frequency (as they should be exactly the same) and the external magnetic field strength, and theoretically determine the wavelength of the measured spin wave through eq. (9). This gives us $\lambda \sim 6\,\mu\text{m}$ for our measurement in fig. 4 of the main text, which agrees reasonably well with our measurement given the imperfect alignment of both the nanobeam and the external magnetic field.

Uncertainty Evaluation of Spin Wave Imaging

In the 1D spin wave imaging measurement shown in fig. 4(c) of the main text, the photoluminescence from the fiber-coupled nanobeam is measured both at the NV ESR frequency $f = 2.439\,\text{GHz}$ and at an off-resonant reference frequency $f_0 = 2.300\,\text{GHz}$, and the ratio of the two counts PL/PL_0 is plotted. For each data point in fig. 4(c), the photoluminescence rates PL and PL_0 are measured intermittently, by switching the microwave frequency between f and f_0 at a fixed interval $\delta T = 0.01\,\text{s}$ for 4000 switching cycles, yielding a total measurement time of $T = 40\,\text{s}$ at each frequency.

Because of the rapid switching, $1/f$ noise is suppressed and we expect photon shot noise to dominate the uncertainty. With the shot noise given by $\Delta(\text{PL}) = \sqrt{\text{PL} \cdot T}$ (same for PL_0), the error bars for the ratio of the photoluminescence rates plotted in fig. 4(c) are calculated using

$$\Delta\left(\frac{\text{PL}}{\text{PL}_0}\right) = \sqrt{\left(\frac{\Delta(\text{PL})}{\text{PL}_0}\right)^2 + \left(\frac{\text{PL}\Delta(\text{PL}_0)}{\text{PL}_0^2}\right)^2}. \tag{17}$$

References

(1) Wee, T. L.; Tzeng, Y. K.; Han, C. C.; Chang, H. C.; Fann, W.; Hsu, J. H.; Chen, K. M.; Yu, E. C. Two-photon excited fluorescence of nitrogen-vacancy centers in proton-irradiated type Ib diamond. *Journal of Physical Chemistry A* **2007**, *111*, 9379–9386.

(2) Rustagi, A.; Bertelli, I.; Van Der Sar, T.; Upadhyaya, P. Sensing chiral magnetic noise via quantum impurity relaxometry. *Physical Review B* **2020**, *102*, 220403.

(3) Dréau, A.; Lesik, M.; Rondin, L.; Spinicelli, P.; Arcizet, O.; Roch, J. F.; Jacques, V. Avoiding power broadening in optically detected magnetic resonance of single NV defects for enhanced dc magnetic field sensitivity. *Physical Review B - Condensed Matter and Materials Physics* **2011**, *84*, 195204.
